# Modulation of Regulatory T Cell Activity by TNF Receptor Type II-Targeting Pharmacological Agents

**DOI:** 10.3389/fimmu.2018.00594

**Published:** 2018-03-26

**Authors:** Huimin Zou, Ruixin Li, Hao Hu, Yuanjia Hu, Xin Chen

**Affiliations:** State Key Laboratory of Quality Research in Chinese Medicine, Institute of Chinese Medical Sciences, University of Macau, Macau, China

**Keywords:** TNF receptor type II, regulatory T cells, TNF receptor type II agonists, TNF receptor type II antagonists, immunotherapy

## Abstract

There is now compelling evidence that tumor necrosis factor (TNF)–TNF receptor type II (TNFR2) interaction plays a decisive role in the activation, expansion, and phenotypical stability of suppressive CD4^+^Foxp3^+^ regulatory T cells (Tregs). In an effort to translate this basic research finding into a therapeutic benefit, a number of agonistic or antagonistic TNFR2-targeting biological agents with the capacity to activate or inhibit Treg activity have been developed and studied. Recent studies also show that thalidomide analogs, cyclophosphamide, and other small molecules are able to act on TNFR2, resulting in the elimination of TNFR2-expressing Tregs. In contrast, pharmacological agents, such as vitamin D3 and adalimumab, were reported to induce the expansion of Tregs by promoting the interaction of transmembrane TNF (tmTNF) with TNFR2. These studies clearly show that TNFR2-targeting pharmacological agents represent an effective approach to modulating the function of Tregs and thus may be useful in the treatment of major human diseases such as autoimmune disorders, graft-versus-host disease (GVHD), and cancer. In this review, we will summarize and discuss the latest progress in the study of TNFR2-targeting pharmacological agents and their therapeutic potential based on upregulation or downregulation of Treg activity.

## Introduction

CD4^+^FoxP3^+^ regulatory T cells (Tregs) play an indispensable role in maintaining immunological homeostasis and inhibiting autoimmune responses, while they also represent a major cellular mechanism in immune evasion of tumors by dampening antitumor immune responses (1, 2). Consequently, Tregs have become important therapeutic target in the treatment of autoimmune diseases, graft-versus-host disease (GVHD), transplantation rejection, and cancer.

We (Xin Chen and Joost J. Oppenheim) previously reported that tumor necrosis factor (TNF)-alpha stimulates the activation and expansion of Tregs, and this effect of TNF is mediated by TNF receptor type II (TNFR2) (3). Moreover, we showed that the expression of TNFR2 correlated with suppressive function and phenotypical stability of Tregs (4–7). Our finding that TNF–TNFR2 interactions play a decisive role in Treg function is now supported by compelling evidence from both human Treg studies (8–24) and mouse Treg studies (25–40) by other groups. Some of these independent studies also clearly show that the Treg-stimulatory effect of TNF–TNFR2 pathway can be therapeutically harnessed for the treatment of major human diseases, including cancer and autoimmune disorders (10, 12, 14, 16, 18, 20, 23, 24).

To translate this basic research finding into therapeutic benefit, a number of agonistic or antagonistic TNFR2-targeting biological agents with the capacity to upregulate or downregulate Treg activity have been developed. Recent study also revealed that some small molecule compounds can suppress TNFR2 expression or eliminate TNFR2-expressing Tregs. Some pharmacological agents were found to induce Tregs by promoting interaction of transmembrane TNF (tmTNF) with TNFR2. In this brief review, recent reports of TNFR2-targeting pharmacological agents with the capacity to upregulate or downregulate Treg activity were reviewed, analyzed, and discussed (Table 1).

**Table 1:** TNF receptor type II (TNFR2)-targeting pharmacological agents.

Category	Class	Agent	Activity	Reference
TNFR2 agonists	Agonistic TNFR2 monoclonal antibodies (mAbs)	“TNFR2 antagonist”	• Binds to and activates human TNFR2 • Stimulates the activation and expansion of homogeneous and highly functional regulatory T cells (Tregs) isolated from normal donors and patients with type 1 diabetes (T1D) (*in vitro* assay)	(10, 18)
		MR2-1 (isotype: IgG1)	• Binds to and activates human TNFR2 • Promotes the expansion of homogenous Foxp3^+^Helios^+^CD127^low^ Treg population with highly suppressive capacity (*in vitro* assay and in humanized mouse study)	(20)
	
	Tumor necrosis factor (TNF) muteins	TNF07	• Binds to and activates human TNFR2 • Expands Foxp3^+^ Treg cells from normal donors (*in vitro* assay) • Selectively induces the death of autoreactive CD8^+^ T cells from T1D patients (*in vitro* assay)	(14)
		STAR2	• Binds to and activates mouse TNFR2 • Stimulates proliferative expansion of Foxp3^+^ Tregs (*in vitro* assay) • Selectively activates and expands Foxp3^+^ Tregs in WT mice (*in vivo* assay) • Markedly prolongs the survival and decreases the severity of graft-versus-host disease (GVHD) (*in vivo* assay)	(38)
		TNC-scTNF(R2)	• Binds to and activates human TNFR2 • Protects TNFR2-expressing oligodendrocyte progenitor cells from death induced by oxidative stress (*in vitro* assay) • Unknown effect on human Tregs	(41)
		EHD2-scTNFR2	• Binds to and activates mouse TNFR2 • Inhibits neuroinflammation and promotes neuronal survival in a mouse model of neurodegeneration in combination with a TNFR1 antagonist (*in vivo* assay) • Unknown effect on mouse Tregs	(42)
	
	Anti-TNF mAbs	Adalimumab	• A therapeutic humanized mAb binding to both soluble TNF (sTNF) and transmembrane TNF (tmTNF) • Increases expression of tmTNF on monocytes from rheumatoid arthritis (RA) patients (*in vitro* assay) • Promotes the binding of tmTNF (expressed on monocytes) to TNFR2 (expressed by Tregs of RA patients), resulting in selective activation and proliferation of Tregs (*in vitro* assay)	(16)
		Infliximab	• A therapeutic humanized mAb against TNF-α • Increases the suppressive function of Tregs in autoimmune patients, at least partially caused by the elevated levels of TNF (*in vivo* assay)	(4, 15, 22)
	
	Small molecule compounds	Vitamin D3	• VD3-DCs induces induced Tregs (iTregs) through the interaction of tmTNF expressed by VD3-DCs and TNFR2 expressed by Tregs (*in vitro* assay)	(26)
TNFR2 antagonists	Antagonistic TNFR2 mAbs	“TNFR2 antagonist”	• Blocks the binding of TNF to human TNFR2 • Markedly inhibits the expansion of Tregs and reduces the suppressive capacity of Tregs (*in vitro* assay)	(10)
		Dominant anti-human TNFR2 antagonistic Abs	• Block the binding of TNF to human TNFR2 and hamper TNFR2 signaling activation • Inhibit TNF-induced expansion of human Tregs (*in vitro* assay) • Induce the death of Tregs, especially those isolated from ovarian cancer tissue (*in vitro* assay) • Induce the death of TNFR2-expressing OVCAR3 tumor cells (*in vitro* assay)	(23)
	
	Small molecule compounds	Thalidomide and its analogs	• Inhibit TNF synthesis • Inhibit the surface expression of TNFR2 on T cells (*in vitro* assay) • Reduce the number and function of Tregs and TNFR2 expression on Tregs in patients with leukemia (*in vivo* assay) • Increase the number of Tregs in patients with multiple myeloma (MM) (*in vivo* assay)	(13, 62, 63, 65, 67, 110)
	
		Panobinostat	• Reduces the expression of Foxp3 and inhibit the suppressive function of Tregs at low doses (*in vitro* assay) • Reduces the proportions of TNFR2^+^ Tregs in the blood and bone marrow of acute myeloid leukemia (AML) patients in combination with azacitidine (*in vivo* assay)	(12, 70)
		Cyclophosphamide	• Selectively depletes TNFR2^hi^ Tregs population in a mouse model of mesothelioma (*in vivo* assay)	(74)
		Triptolide	• Reduces TNF and TNFR2 expression in colon of colitis mice (*in vivo* assay) • Reduces the number of Tregs and inhibits tumor growth in melanoma-bearing mice (*in vivo* assay)	(76, 77)

## TNFR2 Agonistic Biological Agents

Faustman’s group has screened a panel of monoclonal antibodies (mAbs) against human TNFR2 generated from her own lab or purchased from commercial sources. They identified a potent agonistic TNFR2 mAb which was designated as “TNFR2 agonist” in their study. In the presence of IL-2, “TNFR2 agonist” potently stimulated the expansion of Foxp3^+^ Tregs present in cultures of CD4 cells, accompanied by the upregulation of TNF, TRAF2, TRAF3, BIRC3 (cIAP2), and Foxp3 mRNA expression (10). Furthermore, this property of the “TNFR2 agonist” was harnessed to generate highly homogenous Foxp3^+^ Tregs. To this end, MACS-purified CD4^+^CD25^+^ cells were cultured under standard *in vitro* human Treg expansion conditions (anti-CD3 Ab, anti-CD28 Ab, IL-2, and rapamycin), with or without the “TNFR2 agonist.” Expanded Tregs in the presence of “TNFR2 agonist” expressed markedly higher levels of Foxp3 and other characteristic Treg markers, and possessed more potent suppressive capacity (10). More recently, Faustman’s group examined the effect of such “TNFR2 agonist” on the activation and expansion of Tregs isolated from patients with type 1 diabetes (T1D) (18). The results show that *in vitro* treatment with “TNFR2 agonist” stimulated the activation of T1D Tregs which initially showed a resting phenotype. Furthermore, under the aforementioned standard Treg expansion culture condition, “TNFR2 agonist” promoted the homogenous expansion of Tregs isolated from T1D patients by magnetic beads (18). “TNFR2 agonist”-expanded T1D Tregs were more potent in the inhibition of autologous CD8^+^ T cells (18). A similar result was obtained by using MR2-1, a commercially available agonistic human TNFR2 mAb (mouse IgG_1_) by another group (He/Joosten and colleagues) (20). In this study, low purity MACS-isolated human Tregs were expanded with the aforementioned standard protocol. The treatment with MR2-1 resulted in the generation of more homogenous Foxp3^+^Helios^+^CD127^low^ Tregs. The phenotype of resultant Treg cells remained stable, even in the pro-inflammatory environment. Importantly, Tregs expanded with MR2-1 maintained highly suppressive activity in a humanized mouse model (20). Thus, TNFR2 agonists can facilitate *ex vivo* expansion of Treg cells from less pure population for Treg-based immunotherapy.

Prompted by the potential therapeutic effect on autoimmune diseases, Faustman’s group also generated soluble TNF (sTNF) muteins with TNFR2 agonistic effect, designated S95C/G148C or TNF07 (14). This stable TNF trimer, TNF07 double mutant, functioned as a TNFR2 agonist. It could trigger a strong TNFR2 signaling, with the capacity to expand Foxp3^+^ Treg cells and to selectively induce the death of autoreactive CD8^+^ T cells isolated from T1D patients (14).

Chopra/Beilhack and colleagues developed a novel nonameric TNFR2-specific variant of mouse TNF (STAR2), which was a selective agonist of mouse TNFR2 and had no capacity to bind to TNFR1 (38). STAR2 had *in vitro* and *in vivo* activity to stimulate the proliferation of Tregs in a TNFR2-dependent and IL-2-independent manner. Furthermore, pretreatment with STAR2 before allogeneic hematopoietic stem cell transplantation (allo-HCT) markedly prolonged the survival and decreased the severity of GVHD, in TNFR2- and Treg-dependent manner. A human TNFR2-specific STAR2 equivalent agonist also potently stimulated the expansion of Foxp3^+^ Tregs from healthy donors *in vitro* (38).

A number of TNFR2-targeting agents, such as TNC-scTNF(R2) (a human TNFR2 selective agonist) (41) and EHD2-scTNFR2 (a mouse TNFR2 selective agonist) (42), were developed to examine their protective effect on neurodegeneration. It would be interesting to ask if their neuroprotective effect is attributable to their capacity to activate and expand Tregs, and if they have beneficial effect in the inhibition of autoimmune diseases.

It was shown recently that TROS, a nanobody-based selective inhibitor of TNFR1, was able to inhibit mouse experimental autoimmune encephalomyelitis (EAE) and this effect is attributable to the diversion of TNF to interact with TNFR2 (43). TNFR2 is also expressed by oligodendrocytes or astrocytes, with neuroprotective function through tmTNF–TNFR2 signaling to promote CNS cells differentiation and remyelination, and such effect of TNFR2 signaling was based on its directly action on the cells in CNS (44–46). Therefore, selectively blocking TNFR1, thus favoring TNFR2, may represent another strategy to stimulate TNFR2^+^ Tregs in the treatment of autoimmune diseases and GVHD.

## TNFR2 Antagonistic Biological Agents

In addition to a TNFR2 agonist, Faustman’s group also identified a potent mAb antagonist of human TNFR2, designated as “TNFR2 antagonist” in their study (10). In the standard Treg expansion culture condition, this “TNFR2 antagonist” markedly inhibited the expansion of Tregs and reduced the suppressive capacity of Tregs (10). More recently, Torrey/Faustman and colleague developed two potent dominant anti-human TNFR2 antagonistic Abs that outcompeted TNF, the natural agonist of TNFR2, and inhibited TNF-induced *in vitro* expansion of human Tregs (23). These TNFR2 antagonists specifically bound to TNFR2 through F(ab) region, independent of Fc region or crosslinking of antibodies. Through binding to the antiparallel dimers of TNFR2 protein, the TNFR2 antagonists blocked the binding of TNF to TNFR2. Consequently, they inhibited TNF-triggered activation of nuclear factor-κB (NF-κB) pathways in Tregs, and suppressed conversion of tmTNFR2 to sTNFR2. These two TNFR2 antagonists could induce the death of Tregs *in vitro*. Interestingly, Tregs isolated from ovarian cancer tissues were more sensitive to TNFR2 antagonist-induced cell death (23), presumably attributable to the higher levels of TNFR2 expression on tumor-infiltrating Tregs (4). TNFR2 is also expressed on the surface of OVCAR3, an ovarian cancer cell line. Intriguingly, TNFR2 antagonists could also induce the death of OVCAR3 tumor cells (23). Thus, this *in vitro* evidence strongly supports the idea that TNFR2 antagonists may represent novel cancer therapeutics by simultaneously targeting tumor-infiltrating Tregs and tumor cells.

Progranulin (PGRN), a glycosylated protein, has immunosuppressive and anti-inflammatory activity (47–49), presumably due to its capacity to promote the induction of induced Tregs (iTregs), as shown in an *in vitro* study (50). Progranulin was initially reported as an endogenous TNFR2 antagonist (51). However, controversial results were reported (52, 53) and thus further study is needed to clarify its effect on TNFR2.

## Small Molecule TNFR2 Inhibitors

Thalidomide is a synthetic small molecule glutamic acid derivative (54) that was initially developed for alleviation of morning sickness of pregnant women in Europe several decades ago (55). It was withdrawn from the market because it caused developmental defects in newborns (55). The interest in using this compound as a therapeutic agent reawakened recently, due to its suggested effect in the treatment of erythema nodosum leprosum (ENL) (56, 57). This led to the discovery of immunomodulatory and anti-inflammatory properties of thalidomide and to clinical trials of thalidomide and its analogs in various malignancies (54). Thalidomide and its structural analogs (lenalidomide and pomalidomide) are now classified as immunomodulatory drugs (IMiDs) (54). It has been well established that thalidomide and its analogs are able to inhibit TNF protein synthesis through downregulation of NF-κB, destruction of TNF mRNA, and targeting reactive oxygen species and α1-acid glycoprotein (58–61). Thalidomide and its analogs also have the capacity to inhibit the surface expression of TNFR2 on T cells without reducing the expression of total TNFR2 protein (62), which is associated with the inhibition of intracellular TNFR2 transport to the cell surface (13). Giannopoulos et al. showed that, in patients with chronic lymphocytic leukemia, thalidomide treatment reduced the number and function of Tregs (63, 64), presumably by blockade of TNF–TNFR2 interaction. Moreover, Plebanski’s group reported that, in acute myeloid leukemia (AML) patients, combination therapy with lenalidomide and a demethylating agent, azacitidine, downregulated TNFR2 expression on CD4 T cells and reduced the number of TNFR2^+^ Tregs, resulting in enhanced effector immune function (13). However, it was reported that treatment with thalidomide and its analog actually increased the number of Tregs in patients with multiple myeloma (MM) (65, 66), which may be attributable to the elevated serum levels of TNF after treatment (62, 66). Furthermore, thalidomide was reported to promote *de novo* generation of iTregs (67), which is consistent with current understanding of responses of iTreg to TNF–TNFR2 stimulation (29, 68). Thus, the effect of thalidomide on TNFR2^+^ Tregs is likely to be disease- and condition-specific, which should be clarified by future study.

Histone deacetylase inhibitor panobinostat is effective in the treatment of MM in combination with bortezomib and dexamethasone (69). A recent study found that low doses of panobinostat could reduce the expression of Foxp3 and inhibit the suppressive function of Tregs (70). Furthermore, Govindaraj et al. reported that the combination treatment with panobinostat and azacitidine reduced the proportions of TNFR2^+^ Tregs in the blood and bone marrow of AML patients (12). One of the mechanisms may be the disruption of the AML bone marrow niche by panobinostat and azacitidine, resulting in reduced blast cell levels and preventing Treg induction by blast cells (12). The reduction of TNFR2^+^ Tregs and consequently increase of IFNγ and IL-2 production by effector T cells (Teffs) is attributable to the clinical beneficial effect of patients with AML (12). This study indicates that epigenetic therapeutics may represent a strategy to eliminate TNFR2^+^ Treg activity and to enhance antitumor immune responses.

Cyclophosphamide (CY) is a DNA alkylating agent which is commonly used as a cytotoxic chemotherapy in cancer treatment (71). CY at low dosages can inhibit immunosuppressive function of Tregs (72), and a single dose of CY depletes the maximally suppressive Tregs in PROb colon cancer bearing mice, resulting in the activation of antitumor immune responses (73). Moreover, van der Most et al. reported that, in a mouse model of mesothelioma, CY treatment depleted TNFR2^hi^ Tregs (74). This effect of CY was based on its capacity to induce the death of replicating Tregs which co-express TNFR2 and Ki-67 (4, 74). Furthermore, CY in combination with etanercept, a therapeutic TNF antagonist, markedly inhibited the growth of established CT26 tumor in mice, by eliminating TNFR2-expressing Treg activity through blockade of TNF–TNFR2 interaction (75).

Triptolide (TPT), an immunosuppressive compound isolated from Chinese herb *Tripterygium wilfordii* Hook F., was reported to inhibit TNF as well as TNFR2 expression in the colon of mouse colitis model (76). TPT was also reported to decrease the number of Tregs and consequently inhibited the growth of mouse tumor (77). Thus, it would be interesting to investigate if TPT and other naturally occurring compounds have the capacity to downregulate Treg activity by blockade of TNF–TNFR2 interaction.

## Pharmacological Agents that Promote the Interaction of tmTNF and TNFR2

TNF binds and signals through two structurally related functionally distinct receptors: TNFR1 and TNFR2 (78). Once synthesized, TNF is expressed initially as a cell surface type II polypeptide consisting of 233 amino acid residues (26 kDa). Transmembrane TNF is then cleaved by TNF-alpha converting enzyme into a sTNF consisting of 157 amino acid residues (17 kDa) (79). Soluble TNF predominantly binds and activates TNFR1, while tmTNF preferentially binds and activates TNFR2 (80). Therefore, agents which have the capacity to enhance the expression of tmTNF or promote the interaction of tmTNF and TNFR2 may also selectively activate and expand Tregs. This is exemplified by a recent study reported by Nguyen/Ehrenstein showing the paradoxical effect of adalimumab in the expansion of Tregs (16). Adalimumab is a therapeutic anti-TNF mAb which is effective in the treatment of rheumatoid arthritis (RA) and other autoimmune diseases (81). This Ab was developed to bind to both sTNF and tmTNF, aiming to block the interaction of TNF with its receptors (82). It was reported that adalimumab treatment increases the number of Tregs in RA patients (83). A recent *in vitro* study found that adalimumab bound to tmTNF expressed by monocytes from RA patients. This resulted in the upregulation of tmTNF expression, consisting with *in vivo* observations that adalimumab treatment enhanced TNF expression by monocytes from RA patients (16). Furthermore, adalimumab promoted the binding of tmTNF expressed by monocytes to TNFR2 expressed by Tregs of RA patients, consequently enhanced the activation and proliferation of Tregs (16). This study suggests that targeting of tmTNF–TNFR2 interaction may represent a novel strategy in the treatment of autoimmune diseases, especially in those patients that do not to respond to conventional anti-TNF treatment, by mobilization of TNFR2^+^ Tregs (84). Coincidentally, these findings also clarify why adalimumab is more effective in the treatment of Crohn’s disease (85), than etanercept which merely inhibits the effect of sTNF without the concomitant stimulation of Tregs (85, 86).

Infliximab (Remicade) is a therapeutic chimeric mAb against TNF used in the treatment of autoimmune diseases (87). A recent study shows that, in patients with sarcoidosis, surface expression of TNFR2 on CD4^+^CD25^hi^ “Tregs” was higher in responders to therapy, as compared to those non-responders (22). Since TNFR2 expression is associated with suppressive function of Tregs (4, 15), this study suggests that infliximab treatment may also increase the suppressive function of Tregs in autoimmune patients.

It was reported that tolerogenic dendritic cells (DCs), designated as VD3-DCs, were induced by the treatment with 1 alpha, 25-dihydroxyvitamin D3 (VD3). Such DCs expressed high levels of TNF and PD-L1 upon LPS stimulation and were able to induce functionally suppressive Tregs (88). A subsequent study by the same group (Kleijwegt/Roep and colleagues) found that VD3-DCs expressed high levels of tmTNF. Furthermore, induction of Ag-specific Tregs by VD3-DCs depended on the interaction of tmTNF expressed by VD3-DCs and TNFR2 expressed by Tregs, since blockade of binding of tmTNF to TNFR2 abrogated the induction of suppressive function of Tregs (26). In this study, Tregs induced by VD3-DCs were converted from naïve CD4 T cells (26). Thus, the possibility that VD3-DCs can also promote the activation and expansion of naturally occurring Tregs (nTregs) in a tmTNF–TNFR2 dependent manner, especially in the physiologically relevant *in vivo* settings, should be addressed in a future study. Furthermore, since CD8^+^Foxp3^+^ Tregs also expressed high levels of TNFR2 on their surface and TNF signaling is required for the generation of CD8^+^Foxp3^+^ Tregs (89), it would be interesting to investigate if they can be generated or expanded by tmTNF-expressing VD3-DCs.

## Conclusion

Although the first of the TNFR2 inhibitors identified was thalidomide (62), recent research actually focused on the development of TNFR2-targeting biological agents. This may be because the difficulty to block TNF–TNFR interaction with a small molecule, due to the large contact surface area (90), and due to the apparent advantage of biological therapeutics, such as high target specificity, well-understood mechanism and minimal toxicity (91, 92). Nevertheless, cell-permeable small molecules may also effectively block TNFR2 signaling pathways, and consequently inhibit Treg activity induced by TNF–TNFR2 interaction. So far, three signaling pathways of TNFR2 in T lymphocytes, e.g., IKK/NFκB, MAPK (Erk1/2, p38, JNK), and PI3K/Akt pathways, have been reported (93–95). The effect of small molecule inhibitors specific for major components of these pathways on Treg activity should be investigated. Thoroughly understanding of TNFR2 signaling pathways in Tregs, especially those different from Teffs, is a key to identify or design selective Treg inhibitors and thus merits future study. Moreover, it has been shown that TNFR2-specific TNF muteins have the capacity to activate and expand Tregs (38). Since LTα homotrimer can also bind to TNFR2 (96), it would be interesting to investigate if TNFR2-specific mutant LTα have the capacity to preferentially activate Tregs.

In addition to being constitutively and predominantly expressed by highly suppressive Tregs (4), TNFR2 can also be induced and upregulated on CD4^+^Foxp3^−^ Teffs upon TCR stimulation (28, 97). However, the level of TNFR2 expressed by Teffs is much lower than its expression on Tregs (6, 9, 23, 28). This may explain why TNFR2 antibody mimetics preferentially bind to Tregs (21). Nevertheless, TNFR2-targeting agents on the function of Teffs should be carefully evaluated in the future study. Furthermore, in addition to T cells, TNFR2 is also expressed by other cell types, such as endothelial cells (98), microglia and selected neuronal subtypes (99, 100), oligodendrocytes (101), cardiac myocytes (102), and thymocytes (103). Since those TNFR2-expressing cells can also respond to TNFR2-targeting therapeutics, the off-target effect and safety of TNFR2 agonist and antagonist should be carefully evaluated.

**Figure 1:**
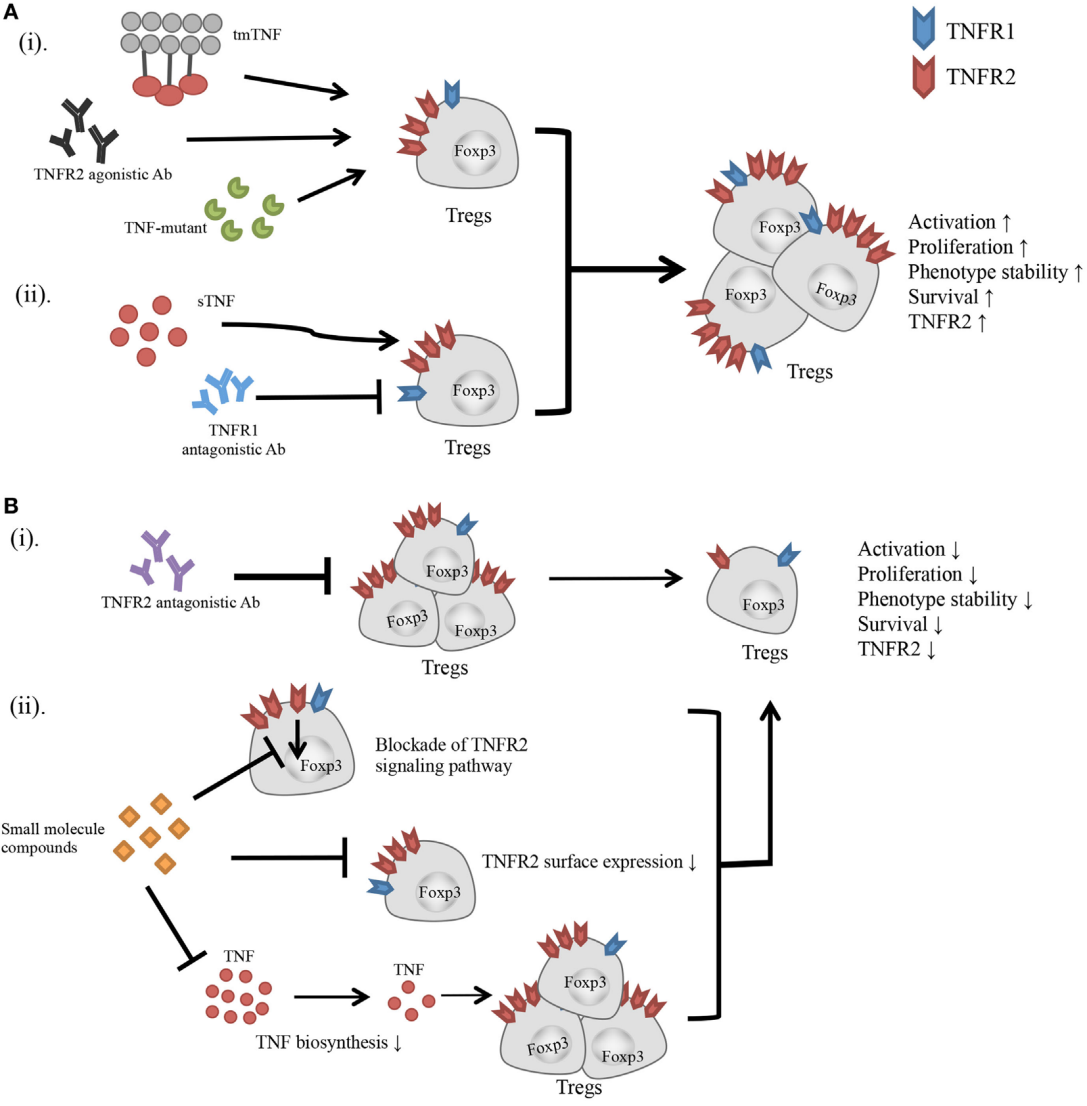
Effect of TNFR2-targeting agents on the activity of Tregs. **(A)** (i) Transmembrane TNF, TNFR2 agonistic Ab, and TNF mutant preferentially bind to and stimulate TNFR2. (ii) Blockade of TNFR1 with antagonistic Ab diverts the stimulatory effect of TNF to TNFR2. All these agents have potential to activate Tregs, and promote the proliferative expansion, phenotypic stability, survival, and TNFR2 expression on Tregs. **(B)** (i) TNFR2 antagonistic Ab blocks TNF–TNFR2 interaction. (ii) Small molecule compounds with the capacity to inhibit TNFR2 signaling pathway, or downregulate TNFR2 surface expression, or suppress TNF biosynthesis. These agents may inhibit the activation and proliferation of Tregs, and reduce the phenotypic stability, survival, and surface TNFR2 expression on Tregs.

Current experimental evidence suggest that TNFR2-targeting agents preferentially act on Tregs, and consequently promote or inhibit immune responses by downregulating or upregulating TNFR2^+^ Treg activity (Figure 1). However, this idea has to be confirmed by more physiologically relevant *in vivo* studies. TNFR2 is also reported to play a key role in the accumulation and immunosuppressive function of myeloid-derived suppressive cells (MDSCs) (34, 104, 105) and mesenchymal stem cells (MSCs) (106, 107). Since these cells exert their immunosuppressive function in a collaborative manner with Tregs (108, 109), the effect of a TNFR2 agonist or antagonist may have a greater effect on the modulation of immune responses, by acting on multiple components of the immunosuppressive network.

Taken together, recent studies regarding TNFR2-targeting agents not only further confirmed and substantiated the concept that TNFR2 signaling plays a decisive role in the activation and expansion of Tregs but they also clearly indicate that TNFR2-targeting pharmacological agents have great potential in the treatment of major human diseases and deserve further research and development.

## Author Contributions

HZ, RL, and XC drafted the manuscript. HZ, RL, HH, YH, and XC approved the final version to be published, and agreed to be accountable for all aspects of the work in ensuring that questions related to the accuracy or integrity of any part of the work are appropriately investigated and resolved.

## Conflict of Interest Statement

The authors declare that the research was conducted in the absence of any commercial or financial relationships that could be construed as a potential conflict of interest.

## References

[1] Sakaguchi S Yamaguchi T Nomura T Ono M. Regulatory T cells and immune tolerance. Cell (2008) 133(5):775–87. 10.1016/j.cell.2008.05.009 18510923

[2] Stephens GL Shevach EM Foxp3+ regulatory T cells: selfishness under scrutiny. Immunity (2007) 27(3):417–9. 10.1016/j.immuni.2007.08.008 17892850

[3] Chen X Baumel M Mannel DN Howard OM Oppenheim JJ. Interaction of TNF with TNF receptor type 2 promotes expansion and function of mouse CD4+CD25+ T regulatory cells. J Immunol (2007) 179(1):154–61. 10.4049/jimmunol.179.1.154 17579033

[4] Chen X Subleski JJ Kopf H Howard OM Mannel DN Oppenheim JJ. Cutting edge: expression of TNFR2 defines a maximally suppressive subset of mouse CD4+CD25+FoxP3+ T regulatory cells: applicability to tumor-infiltrating T regulatory cells. J Immunol (2008) 180(10):6467–71. 10.4049/jimmunol.180.10.6467 18453563 PMC2699949

[5] Chen X Subleski JJ Hamano R Howard OM Wiltrout RH Oppenheim JJ. Co-expression of TNFR2 and CD25 identifies more of the functional CD4+FOXP3+ regulatory T cells in human peripheral blood. Eur J Immunol (2010) 40(4):1099–106. 10.1002/eji.200940022 20127680 PMC3096013

[6] Chen X Wu X Zhou Q Howard OM Netea MG Oppenheim JJ. TNFR2 is critical for the stabilization of the CD4+Foxp3+ regulatory T. cell phenotype in the inflammatory environment. J Immunol (2013) 190(3):1076–84. 10.4049/jimmunol.1202659 23277487 PMC3552130

[7] Chen X Willette-Brown J Wu X Hu Y Howard OM Oppenheim JJ IKKalpha is required for the homeostasis of regulatory T cells and for the expansion of both regulatory and effector CD4 T cells. FASEB J (2015) 29(2):443–54. 10.1096/fj.14-259564 25376833 PMC4314223

[8] Mougiakakos D Johansson CC Jitschin R Bottcher M Kiessling R Increased thioredoxin-1 production in human naturally occurring regulatory T cells confers enhanced tolerance to oxidative stress. Blood (2011) 117(3):857–61. 10.1182/blood-2010-09-307041 21030559

[9] Govindaraj C Scalzo-Inguanti K Madondo M Hallo J Flanagan K Quinn M Impaired Th1 immunity in ovarian cancer patients is mediated by TNFR2+Tregs within the tumor microenvironment. Clin Immunol (2013) 149(1):97–110. 10.1016/j.clim.2013.07.003 23948613

[10] Okubo Y Mera T Wang LM Faustman DL. Homogeneous expansion of human T-regulatory cells via tumor necrosis factor receptor 2. Sci Rep (2013) 3:3153. 10.1038/srep03153 24193319 PMC3818650

[11] Wammes LJ Wiria AE Toenhake CG Hamid F Liu KY Suryani H Asymptomatic plasmodial infection is associated with increased tumor necrosis factor receptor II-expressing regulatory T cells and suppressed type 2 immune responses. J Infect Dis (2013) 207(10):1590–9. 10.1093/infdis/jit058 23408847

[12] Govindaraj C Tan P Walker P Wei A Spencer A Plebanski M. Reducing TNF receptor 2+ regulatory T cells via the combined action of azacitidine and the HDAC inhibitor, panobinostat for clinical benefit in acute myeloid leukemia patients. Clin Cancer Res (2014) 20(3):724–35. 10.1158/1078-0432.CCR-13-1576 24297862

[13] Govindaraj C Madondo M Kong YY Tan P Wei A Plebanski M. Lenalidomide-based maintenance therapy reduces TNF receptor 2 on CD4 T cells and enhances immune effector function in acute myeloid leukemia patients. Am J Hematol (2014) 89(8):795–802. 10.1002/ajh.23746 24757092

[14] Ban L Kuhtreiber W Butterworth J Okubo Y Vanamee ES Faustman DL. Strategic internal covalent cross-linking of TNF produces a stable TNF trimer with improved TNFR2 signaling. Mol Cell Ther (2015) 3:7. 10.1186/s40591-015-0044-4 26266038 PMC4531505

[15] Yan F Du R Wei F Zhao H Yu J Wang C Expression of TNFR2 by regulatory T cells in peripheral blood is correlated with clinical pathology of lung cancer patients. Cancer Immunol Immunother (2015) 64(11):1475–85. 10.1007/s00262-015-1751-z 26280204 PMC11029166

[16] Nguyen DX Ehrenstein MR Anti-TNF drives regulatory T cell expansion by paradoxically promoting membrane TNF-TNF-RII binding in rheumatoid arthritis. J Exp Med (2016) 213(7):1241–53. 10.1084/jem.20151255 27270893 PMC4925013

[17] Nguyen MT Fryml E Sahakian SK Liu S Cantarovich M Lipman M Pretransplant recipient circulating CD4+CD127lo/- tumor necrosis factor receptor 2+ regulatory T cells: a surrogate of regulatory T cell-suppressive function and predictor of delayed and slow graft function after kidney transplantation. Transplantation (2016) 100(2):314–24. 10.1097/TP.0000000000000942 26425877

[18] Okubo Y Torrey H Butterworth J Zheng H Faustman DL. Treg activation defect in type 1 diabetes: correction with TNFR2 agonism. Clin Transl Immunology (2016) 5:e56. 10.1038/cti.2015.43 26900470 PMC4735064

[19] Zaragoza B Chen X Oppenheim JJ Baeyens A Gregoire S Chader D Suppressive activity of human regulatory T cells is maintained in the presence of TNF. Nat Med (2016) 22(1):16–7. 10.1038/nm.4019 26735402 PMC6345394

[20] He XH Landman S Bauland SCG van den Dolder J Koenen HJPM Joosten I. A TNFR2-agonist facilitates high purity expansion of human low purity Treg cells. PLoS One (2016) 11(5):e0156311. 10.1371/journal.pone.0156311 27224512 PMC4880213

[21] Williams GS Mistry B Guillard S Ulrichsen JC Sandercock AM Wang J Phenotypic screening reveals TNFR2 as a promising target for cancer immunotherapy. Oncotarget (2016) 7(42):68278–91. 10.18632/oncotarget.11943 27626702 PMC5356554

[22] Verwoerd A Hijdra D Vorselaars AD Crommelin HA van Moorsel CH Grutters JC Infliximab therapy balances regulatory T cells, tumour necrosis factor receptor 2 (TNFR2) expression and soluble TNFR2 in sarcoidosis. Clin Exp Immunol (2016) 185(2):263–70. 10.1111/cei.12808 27158798 PMC4955009

[23] Torrey H Butterworth J Mera T Okubo Y Wang L Baum D Targeting TNFR2 with antagonistic antibodies inhibits proliferation of ovarian cancer cells and tumor-associated Tregs. Sci Signal (2017) 10(462):eaaf8608. 10.1126/scisignal.aaf8608 28096513

[24] Ferrarelli LK Locking TNFR2 to kill ovarian cancer. Science (2017) 355(6322):257–8. 10.1126/science.355.6322.257-h 28104869

[25] van Mierlo GJ Scherer HU Hameetman M Morgan ME Flierman R Huizinga TW Cutting edge: TNFR-shedding by CD4+CD25+ regulatory T cells inhibits the induction of inflammatory mediators. J Immunol (2008) 180(5):2747–51. 10.4049/jimmunol.180.5.2747 18292492

[26] Kleijwegt FS Laban S Duinkerken G Joosten AM Zaldumbide A Nikolic T Critical role for TNF in the induction of human antigen-specific regulatory T cells by tolerogenic dendritic cells. J Immunol (2010) 185(3):1412–8. 10.4049/jimmunol.1000560 20574005

[27] Grinberg-Bleyer Y Saadoun D Baeyens A Billiard F Goldstein JD Gregoire S Pathogenic T cells have a paradoxical protective effect in murine autoimmune diabetes by boosting Tregs. J Clin Invest (2010) 120(12):4558–68. 10.1172/JCI42945 21099113 PMC2993590

[28] Chen X Hamano R Subleski JJ Hurwitz AA Howard OM Oppenheim JJ. Expression of costimulatory TNFR2 induces resistance of CD4+FoxP3- conventional T cells to suppression by CD4+FoxP3+ regulatory T cells. J Immunol (2010) 185(1):174–82. 10.4049/jimmunol.0903548 20525892 PMC6314668

[29] Housley WJ Adams CO Nichols FC Puddington L Lingenheld EG Zhu L Natural but not inducible regulatory T cells require TNF-alpha signaling for in vivo function. J Immunol (2011) 186(12):6779–87. 10.4049/jimmunol.1003868 21572024

[30] Chopra M Riedel SS Biehl M Krieger S von Krosigk V Bauerlein CA Tumor necrosis factor receptor 2-dependent homeostasis of regulatory T cells as a player in TNF-induced experimental metastasis. Carcinogenesis (2013) 34(6):1296–303. 10.1093/carcin/bgt038 23385062

[31] Myers L Joedicke JJ Carmody AB Messer RJ Kassiotis G Dudley JP IL-2-independent and TNF-alpha-dependent expansion of Vbeta5+ natural regulatory T cells during retrovirus infection. J Immunol (2013) 190(11):5485–95. 10.4049/jimmunol.1202951 23645880 PMC3739475

[32] Mahmud SA Manlove LS Schmitz HM Xing Y Wang Y Owen DL Costimulation via the tumor-necrosis factor receptor superfamily couples TCR signal strength to the thymic differentiation of regulatory T cells. Nat Immunol (2014) 15(5):473–81. 10.1038/ni.2849 24633226 PMC4000541

[33] Joedicke JJ Myers L Carmody AB Messer RJ Wajant H Lang KS Activated CD8+ T cells induce expansion of Vbeta5+ regulatory T cells via TNFR2 signaling. J Immunol (2014) 193(6):2952–60. 10.4049/jimmunol.1400649 25098294 PMC4157120

[34] Ham B Wang N D’Costa Z Fernandez MC Bourdeau F Auguste P TNF receptor-2 facilitates an immunosuppressive microenvironment in the liver to promote the colonization and growth of hepatic metastases. Cancer Res (2015) 75(24):5235–47. 10.1158/0008-5472.CAN-14-3173 26483205

[35] Baeyens A Saadoun D Billiard F Rouers A Gregoire S Zaragoza B Effector T cells boost regulatory T cell expansion by IL-2, TNF, OX40, and plasmacytoid dendritic cells depending on the immune context. J Immunol (2015) 194(3):999–1010. 10.4049/jimmunol.1400504 25548233

[36] Leclerc M Naserian S Pilon C Thiolat A Martin GH Pouchy C Control of GVHD by regulatory T cells depends on TNF produced by T cells and TNFR2 expressed by regulatory T cells. Blood (2016) 128(12):1651–9. 10.1182/blood-2016-02-700849 27506541

[37] Pierini A Strober W Moffett C Baker J Nishikii H Alvarez M TNF-alpha priming enhances CD4+FoxP3+ regulatory T-cell suppressive function in murine GVHD prevention and treatment. Blood (2016) 128(6):866–71. 10.1182/blood-2016-04-711275 27365424 PMC4982455

[38] Chopra M Biehl M Steinfatt T Brandl A Kums J Amich J Exogenous TNFR2 activation protects from acute GvHD via host Treg cell expansion. J Exp Med (2016) 213(9):1881–900. 10.1084/jem.20151563 27526711 PMC4995078

[39] Krummey SM Chen CW Guasch SA Liu D Wagener M Larsen CP Enhanced requirement for TNFR2 in graft rejection mediated by low-affinity memory CD8+ T cells during heterologous immunity. J Immunol (2016) 197(5):2009–15. 10.4049/jimmunol.1502680 27481849 PMC4992585

[40] Schmidt D Peterlik D Reber SO Lechner A Mannel DN Induction of suppressor cells and increased tumor growth following chronic psychosocial stress in male mice. PLoS One (2016) 11(7):e0159059 10.1371/journal.pone.0159059 27391954 PMC4938385

[41] Maier O Fischer R Agresti C Pfizenmaier K. TNF receptor 2 protects oligodendrocyte progenitor cells against oxidative stress. Biochem Biophys Res Commun (2013) 440(2):336–41. 10.1016/j.bbrc.2013.09.083 24076392

[42] Dong Y Fischer R Naude PJ Maier O Nyakas C Duffey M Essential protective role of tumor necrosis factor receptor 2 in neurodegeneration. Proc Natl Acad Sci U S A (2016) 113(43):12304–9. 10.1073/pnas.1605195113 27791020 PMC5087045

[43] Steeland S Van Ryckeghem S Van Imschoot G De Rycke R Toussaint W Vanhoutte L TNFR1 inhibition with a nanobody protects against EAE development in mice. Sci Rep (2017) 7(1):13646 10.1038/s41598-017-13984-y 29057962 PMC5651799

[44] Madsen PM Motti D Karmally S Szymkowski DE Lambertsen KL Bethea JR Oligodendroglial TNFR2 mediates membrane TNF-dependent repair in experimental autoimmune encephalomyelitis by promoting oligodendrocyte differentiation and remyelination. J Neurosci (2016) 36(18):5128–43. 10.1523/jneurosci.0211-16.2016 27147664 PMC4854972

[45] Fischer R Wajant H Kontermann R Pfizenmaier K Maier O. Astrocyte-specific activation of TNFR2 promotes oligodendrocyte maturation by secretion of leukemia inhibitory factor. Glia (2014) 62(2):272–83. 10.1002/glia.22605 24310780

[46] Taoufik E Tseveleki V Chu SY Tselios T Karin M Lassmann H Transmembrane tumour necrosis factor is neuroprotective and regulates experimental autoimmune encephalomyelitis via neuronal nuclear factor-kappa B. Brain (2011) 134:2722–35. 10.1093/brain/awr203 21908876

[47] Martens LH Zhang JS Barmada SJ Zhou P Kamiya S Sun BG Progranulin deficiency promotes neuroinflammation and neuron loss following toxin-induced injury. J Clin Invest (2012) 122(11):3955–9. 10.1172/Jci63113 23041626 PMC3484443

[48] Zhao YP Tian QY Liu CJ. Progranulin deficiency exaggerates, whereas progranulin-derived Atsttrin attenuates, severity of dermatitis in mice. FEBS Lett (2013) 587(12):1805–10. 10.1016/j.febslet.2013.04.037 23669357 PMC3683372

[49] Bossu P Salani F Alberici A Archetti S Bellelli G Galimberti D Loss of function mutations in the progranulin gene are related to pro-inflammatory cytokine dysregulation in frontotemporal lobar degeneration patients. J Neuroinflammation (2011) 8:65. 10.1186/1742-2094-8-65 21645364 PMC3141503

[50] Wei F Zhang Y Zhao W Yu X Liu CJ Progranulin facilitates conversion and function of regulatory T cells under inflammatory conditions. PLoS One (2014) 9(11):e112110 10.1371/journal.pone.0112110 25393765 PMC4230946

[51] Tang W Lu Y Tian QY Zhang Y Guo FJ Liu GY The growth factor progranulin binds to TNF receptors and is therapeutic against inflammatory arthritis in mice. Science (2011) 332(6028):478–84. 10.1126/science.1199214 21393509 PMC3104397

[52] Hu Y Xiao H Shi T Oppenheim JJ Chen X Progranulin promotes tumour necrosis factor-induced proliferation of suppressive mouse CD4(+) Foxp3(+) regulatory T cells. Immunology (2014) 142(2):193–201. 10.1111/imm.12241 24383743 PMC4008227

[53] Chen X Chang J Deng Q Xu J Nguyen TA Martens LH Progranulin does not bind tumor necrosis factor (TNF) receptors and is not a direct regulator of TNF-dependent signaling or bioactivity in immune or neuronal cells. J Neurosci (2013) 33(21):9202–13. 10.1523/JNEUROSCI.5336-12.2013 23699531 PMC3707136

[54] Shortt J Hsu AK Johnstone RW. Thalidomide-analogue biology: immunological, molecular and epigenetic targets in cancer therapy. Oncogene (2013) 32(36):4191–202. 10.1038/onc.2012.599 23318436

[55] Palumbo A Facon T Sonneveld P Blade J Offidani M Gay F Thalidomide for treatment of multiple myeloma: 10 years later. Blood (2008) 111(8):3968–77. 10.1182/blood-2007-10-117457 18245666

[56] Sheskin J Thalidomide in the treatment of lepra reactions. Clin Pharmacol Ther (1965) 6:303–6. 10.1002/cpt196563303 14296027

[57] Iyer CG Languillon J Ramanujam K Tarabini-Castellani G De las Aguas JT Bechelli LM WHO co-ordinated short-term double-blind trial with thalidomide in the treatment of acute lepra reactions in male lepromatous patients. Bull World Health Organ (1971) 45(6):719–32. 4947831 PMC2427977

[58] Moreira AL Sampaio EP Zmuidzinas A Frindt P Smith KA Kaplan G. Thalidomide exerts its inhibitory action on tumor necrosis factor alpha by enhancing mRNA degradation. J Exp Med (1993) 177(6):1675–80. 10.1084/jem.177.6.1675 8496685 PMC2191046

[59] Turk BE Jiang H Liu JO Binding of thalidomide to alpha1-acid glycoprotein may be involved in its inhibition of tumor necrosis factor alpha production. Proc Natl Acad Sci U S A (1996) 93(15):7552–6. 10.1073/pnas.93.15.7552 8755512 PMC38783

[60] Paul SC Lv P Xiao YJ An P Liu SQ Luo HS. Thalidomide in rat liver cirrhosis: blockade of tumor necrosis factor-alpha via inhibition of degradation of an inhibitor of nuclear factor-kappaB. Pathobiology (2006) 73(2):82–92. 10.1159/000094492 16943688

[61] Majumder S Sreedhara SR Banerjee S Chatterjee S TNF alpha signaling beholds thalidomide saga: a review of mechanistic role of TNF-alpha signaling under thalidomide. Curr Top Med Chem (2012) 12(13):1456–67. 10.2174/156802612801784443 22650377

[62] Marriott JB Clarke IA Dredge K Muller G Stirling D Dalgleish AG Thalidomide and its analogues have distinct and opposing effects on TNF-α and TNFR2 during co-stimulation of both CD4+ and CD8+ T cells. Clin Exp Immunol (2002) 130(1):75–84. 10.1046/j.1365-2249.2002.01954.x 12296856 PMC1906488

[63] Giannopoulos K Dmoszynska A Kowal M Wasik-Szczepanek E Bojarska-Junak A Rolinski J Thalidomide exerts distinct molecular antileukemic effects and combined thalidomide/fludarabine therapy is clinically effective in high-risk chronic lymphocytic leukemia. Leukemia (2009) 23(10):1771–8. 10.1038/leu.2009.98 19440214

[64] Giannopoulos K Schmitt M Wlasiuk P Chen J Bojarska-Junak A Kowal M The high frequency of T regulatory cells in patients with B-cell chronic lymphocytic leukemia is diminished through treatment with thalidomide. Leukemia (2008) 22(1):222–4. 10.1038/sj.leu.2404869 17657216

[65] Gupta R Ganeshan P Hakim M Verma R Sharma A Kumar L Significantly reduced regulatory T cell population in patients with untreated multiple myeloma. Leuk Res (2011) 35(7):874–8. 10.1016/j.leukres.2010.11.010 21146215

[66] Quach H Ritchie D Neeson P Harrison S Tai T Tainton K Regulatory T cells (Treg) are depressed in patients with relapsed/refractory multiple myeloma (MM) and increases towards normal range in responding patients treated with lenalidomide (LEN). Blood (2008) 112(11):599.

[67] Gori A Rossi MC Trabattoni D Marchetti G Fusi ML Molteni C Tumor necrosis factor-alpha increased production during thalidomide treatment in patients with tuberculosis and human immunodeficiency virus coinfection. J Infect Dis (2000) 182(2):639–40. 10.1086/315721 10915104

[68] Zhang Q Cui FF Fang L Hong J Zheng BA Zhang JWZ TNF- impairs differentiation and function of TGF – induced Treg cells in autoimmune diseases through Akt and Smad3 signaling pathway. J Mol Cell Biol (2013) 5(2):85–98. 10.1093/jmcb/mjs063 23243069

[69] Laubach JP Moreau P San-Miguel JF Richardson PG. Panobinostat for the treatment of multiple myeloma. Clin Cancer Res (2015) 21(21):4767–73. 10.1158/1078-0432.CCR-15-0530 26362997

[70] Shen L Pili R Class I histone deacetylase inhibition is a novel mechanism to target regulatory T cells in immunotherapy. Oncoimmunology (2012) 1(6):948–50. 10.4161/onci.20306 23162767 PMC3489755

[71] Emadi A Jones RJ Brodsky RA. Cyclophosphamide and cancer: golden anniversary. Nat Rev Clin Oncol (2009) 6(11):638–47. 10.1038/nrclinonc.2009.146 19786984

[72] Lutsiak ME Semnani RT De Pascalis R Kashmiri SV Schlom J Sabzevari H Inhibition of CD4(+)25+ T regulatory cell function implicated in enhanced immune response by low-dose cyclophosphamide. Blood (2005) 105(7):2862–8. 10.1182/blood-2004-06-2410 15591121

[73] Ghiringhelli F Larmonier N Schmitt E Parcellier A Cathelin D Garrido C CD4(+)CD25(+) regulatory T cells suppress tumor immunity but are sensitive to cyclophosphamide which allows immunotherapy of established tumors to be curative. Eur J Immunol (2004) 34(2):336–44. 10.1002/eji.200324181 14768038

[74] van der Most RG Currie AJ Mahendran S Prosser A Darabi A Robinson BW Tumor eradication after cyclophosphamide depends on concurrent depletion of regulatory T cells: a role for cycling TNFR2-expressing effector-suppressor T cells in limiting effective chemotherapy. Cancer Immunol Immunother (2009) 58(8):1219–28. 10.1007/s00262-008-0628-9 19052741 PMC11030690

[75] Chang LY Lin YC Chiang JM Mahalingam J Su SH Huang CT Blockade of TNF-alpha signaling benefits cancer therapy by suppressing effector regulatory T cell expansion. Oncoimmunology (2015) 4(10):e1040215 10.1080/2162402X.2015.1040215 26451304 PMC4589045

[76] Wei X Gong J Zhu J Wang P Li N Zhu W The suppressive effect of triptolide on chronic colitis and TNF-alpha/TNFR2 signal pathway in interleukin-10 deficient mice. Clin Immunol (2008) 129(2):211–8. 10.1016/j.clim.2008.07.018 18757245

[77] Liu B Zhang H Li J Lu C Chen G Zhang G Triptolide downregulates Treg cells and the level of IL-10, TGF-beta, and VEGF in melanoma-bearing mice. Planta Med (2013) 79(15):1401–7. 10.1055/s-0033-1350708 23975869

[78] Vandenabeele P Declercq W Beyaert R Fiers W Two tumour necrosis factor receptors: structure and function. Trends Cell Biol (1995) 5(10):392–9. 10.1016/S0962-8924(00)89088-1 14732063

[79] Black RA Rauch CT Kozlosky CJ Peschon JJ Slack JL Wolfson MF A metalloproteinase disintegrin that releases tumour-necrosis factor-alpha from cells. Nature (1997) 385(6618):729–33. 10.1038/385729a0 9034190

[80] Grell M Douni E Wajant H Lohden M Clauss M Maxeiner B The transmembrane form of tumor necrosis factor is the prime activating ligand of the 80 kDa tumor necrosis factor receptor. Cell (1995) 83(5):793–802. 10.1016/0092-8674(95)90192-2 8521496

[81] Lapadula G Marchesoni A Armuzzi A Blandizzi C Caporali R Chimenti S Adalimumab in the treatment of immune-mediated diseases. Int J Immunopathol Pharmacol (2014) 27(1 Suppl):33–48. 10.1177/03946320140270S103 24774505

[82] Keystone E Haraoui B Adalimumab therapy in rheumatoid arthritis. Rheum Dis Clin North Am (2004) 30(2):349–64. 10.1016/j.rdc.2004.02.004 15172045

[83] McGovern JL Nguyen DX Notley CA Mauri C Isenberg DA Ehrenstein MR. Th17 cells are restrained by Treg cells via the inhibition of interleukin-6 in patients with rheumatoid arthritis responding to anti-tumor necrosis factor antibody therapy. Arthritis Rheum (2012) 64(10):3129–38. 10.1002/art.34565 22674488

[84] Chen X Oppenheim JJ Therapy: paradoxical effects of targeting TNF signalling in the treatment of autoimmunity. Nat Rev Rheumatol (2016) 12(11):625–6. 10.1038/nrrheum.2016.145 27586383 PMC8502420

[85] Tracey D Klareskog L Sasso EH Salfeld JG Tak PP Tumor necrosis factor antagonist mechanisms of action: a comprehensive review. Pharmacol Ther (2008) 117(2):244–79. 10.1016/j.pharmthera.2007.10.001 18155297

[86] Sandborn WJ Hanauer SB Katz S Safdi M Wolf DG Baerg RD Etanercept for active Crohn’s disease: a randomized, double-blind, placebo-controlled trial. Gastroenterology (2001) 121(5):1088–94. 10.1053/gast.2001.28674 11677200

[87] Keane J Gershon S Wise RP Mirabile-Levens E Kasznica J Schwieterman WD Tuberculosis associated with infliximab, a tumor necrosis factor (alpha)-neutralizing agent. N Engl J Med (2001) 345(15):1098–104. 10.1056/NEJMoa011110 11596589

[88] Unger WW Laban S Kleijwegt FS van der Slik AR Roep BO. Induction of Treg by monocyte-derived DC modulated by vitamin D3 or dexamethasone: differential role for PD-L1. Eur J Immunol (2009) 39(11):3147–59. 10.1002/eji.200839103 19688742

[89] Horwitz DA Pan S Ou JN Wang J Chen M Gray JD Therapeutic polyclonal human CD8+ CD25+ Fox3+ TNFR2+ PD-L1+ regulatory cells induced ex-vivo. Clin Immunol (2013) 149(3):450–63. 10.1016/j.clim.2013.08.007 24211847 PMC3941976

[90] Richmond V Michelini FM Bueno CA Alche LE Ramirez JA Small molecules as anti-TNF drugs. Curr Med Chem (2015) 22(25):2920–42. 10.2174/0929867322666150729115553 26219390

[91] Willrich MAV Murray DL Snyder MR. Tumor necrosis factor inhibitors: clinical utility in autoimmune diseases. Transl Res (2015) 165(2):270–82. 10.1016/j.trsl.2014.09.006 25305470

[92] Monaco C Nanchahal J Taylor P Feldmann M. Anti-TNF therapy: past, present and future. Int Immunol (2015) 27(1):55–62. 10.1093/intimm/dxu102 25411043 PMC4279876

[93] Faustman D Davis M. TNF receptor 2 pathway: drug target for autoimmune diseases. Nat Rev Drug Discov (2010) 9(6):482–93. 10.1038/nrd3030 20489699

[94] Kim EY Priatel JJ Teh SJ Teh HS. TNF receptor type 2 (p75) functions as a costimulator for antigen-driven T cell responses in vivo. J Immunol (2006) 176(2):1026–35. 10.4049/jimmunol.176.2.1026 16393990

[95] So T Croft M. Regulation of PI-3-kinase and Akt signaling in T lymphocytes and other cells by TNFR family molecules. Front Immunol (2013) 4:139. 10.3389/fimmu.2013.00139 23760533 PMC3675380

[96] Browning JL Ngam-ek A Lawton P DeMarinis J Tizard R Chow EP Lymphotoxin beta, a novel member of the TNF family that forms a heteromeric complex with lymphotoxin on the cell surface. Cell (1993) 72(6):847–56. 10.1016/0092-8674(93)90574-A 7916655

[97] Govindaraj C Scalzo-Inguanti K Scholzen A Li S Plebanski M TNFR2 expression on CD25(hi)FOXP3(+) T cells induced upon TCR stimulation of CD4 T cells identifies maximal cytokine-producing effectors. Front Immunol (2013) 4:233 10.3389/fimmu.2013.00233 23964278 PMC3734366

[98] Pan S An P Zhang R He XR Yin GY Min W. Etk/Bmx as a tumor necrosis factor receptor type 2-specific kinase: role in endothelial cell migration and angiogenesis. Mol Cell Biol (2002) 22(21):7512–23. 10.1128/mcb.22.21.7512-7523.2002 12370298 PMC135657

[99] Yang LB Lindholm K Konishi Y Li R Shen Y. Target depletion of distinct tumor necrosis factor receptor subtypes reveals hippocampal neuron death and survival through different signal transduction pathways. J Neurosci (2002) 22(8):3025–32. 11943805 10.1523/JNEUROSCI.22-08-03025.2002 PMC6757531

[100] Mccoy MK Tansey MG. TNF signaling inhibition in the CNS: implications for normal brain function and neurodegenerative disease. J Neuroinflammation (2008) 5:45. 10.1186/1742-2094-5-45 18925972 PMC2577641

[101] Arnett HA Mason J Marino M Suzuki K Matsushima GK Ting JPY. TNF alpha promotes proliferation of oligodendrocyte progenitors and remyelination. Nat Neurosci (2001) 4(11):1116–22. 10.1038/nn738 11600888

[102] Irwin MW Mak S Mann DL Qu R Penninger JM Yan A Tissue expression and immunolocalization of tumor necrosis factor-alpha in postinfarction dysfunctional myocardium. Circulation (1999) 99(11):1492–8. 10.1161/01.CIR.99.11.1492 10086975

[103] Annunziato F Cosmi L Liotta F Lazzeri E Manetti R Vanini V Phenotype, localization, and mechanism of suppression of CD4(+)CD25(+) human thymocytes. J Exp Med (2002) 196(3):379–87. 10.1084/jem.20020110 12163566 PMC2193942

[104] Pastille E Bardini K Fleissner D Adamczyk A Frede A Wadwa M Transient ablation of regulatory T cells improves antitumor immunity in colitis-associated colon cancer. Cancer Res (2014) 74(16):4258–69. 10.1158/0008-5472.CAN-13-3065 24906621

[105] Zhao X Rong L Zhao X Li X Liu X Deng J TNF signaling drives myeloid-derived suppressor cell accumulation. J Clin Invest (2012) 122(11):4094–104. 10.1172/JCI64115 23064360 PMC3484453

[106] Chen X Oppenheim JJ. Targeting TNFR2, an immune checkpoint stimulator and oncoprotein, is a promising treatment for cancer. Sci Signal (2017) 10(462):eaal2328. 10.1126/scisignal.aal2328 28096506

[107] Kelly ML Wang M Crisostomo PR Abarbanell AM Herrmann JL Weil BR TNF receptor 2, not TNF receptor 1, enhances mesenchymal stem cell-mediated cardiac protection following acute ischemia. Shock (2010) 33(6):602–7. 10.1097/SHK.0b013e3181cc0913 19953003 PMC3076044

[108] Fujimura T Kambayashi Y Aiba S Crosstalk between regulatory T cells (Tregs) and myeloid derived suppressor cells (MDSCs) during melanoma growth. Oncoimmunology (2012) 1(8):1433–4. 10.4161/onci.21176 23243619 PMC3518528

[109] Miyagawa I Nakayamada S Nakano K Yamagata K Sakata K Yamaoka K Induction of regulatory T cells and its regulation with insulin-like growth factor/insulin-like growth factor binding protein-4 by human mesenchymal stem cells. J Immunol (2017) 199(5):1616–25. 10.4049/jimmunol.1600230 28724578

[110] Skorka K Bhattacharya N Wlasiuk P Kowal M Mertens D Dmoszynska A Thalidomide regulation of NF-kappaB proteins limits Tregs activity in chronic lymphocytic leukemia. Adv Clin Exp Med (2014) 23(1):25–32. 10.17219/acem/37018 24596000

